# Validity of Wrist-Wearable Activity Devices for Estimating Physical Activity in Adolescents: Comparative Study

**DOI:** 10.2196/18320

**Published:** 2021-01-07

**Authors:** Yingying Hao, Xiao-Kai Ma, Zheng Zhu, Zhen-Bo Cao

**Affiliations:** 1 School of Kinesiology Shanghai University of Sport Shanghai China

**Keywords:** wrist-wearable activity devices, accelerometer, energy expenditure, step counts, free-living

## Abstract

**Background:**

The rapid advancements in science and technology of wrist-wearable activity devices offer considerable potential for clinical applications. Self-monitoring of physical activity (PA) with activity devices is helpful to improve the PA levels of adolescents. However, knowing the accuracy of activity devices in adolescents is necessary to identify current levels of PA and assess the effectiveness of intervention programs designed to increase PA.

**Objective:**

The study aimed to determine the validity of the 11 commercially available wrist-wearable activity devices for monitoring total steps and total 24-hour total energy expenditure (TEE) in healthy adolescents under simulated free-living conditions.

**Methods:**

Nineteen (10 male and 9 female) participants aged 14 to 18 years performed a 24-hour activity cycle in a metabolic chamber. Each participant simultaneously wore 11 commercial wrist-wearable activity devices (Mi Band 2 [XiaoMi], B2 [Huawei], Bong 2s [Meizu], Amazfit [Huamei], Flex [Fitbit], UP3 [Jawbone], Shine 2 [Misfit], GOLiFE Care-X [GoYourLife], Pulse O2 [Withings], Vivofit [Garmin], and Loop [Polar Electro]) and one research-based triaxial accelerometer (GT3X+ [ActiGraph]). Criterion measures were total EE from the metabolic chamber (mcTEE) and total steps from the GT3X+ (AGsteps).

**Results:**

Pearson correlation coefficients r for 24-hour TEE ranged from .78 (Shine 2, Amazfit) to .96 (Loop) and for steps ranged from 0.20 (GOLiFE) to 0.57 (Vivofit). Mean absolute percent error (MAPE) for TEE ranged from 5.7% (Mi Band 2) to 26.4% (Amazfit) and for steps ranged from 14.2% (Bong 2s) to 27.6% (Loop). TEE estimates from the Mi Band 2, UP3, Vivofit, and Bong 2s were equivalent to mcTEE. Total steps from the Bong 2s were equivalent to AGsteps.

**Conclusions:**

Overall, the Bong 2s had the best accuracy for estimating TEE and total steps under simulated free-living conditions. Further research is needed to examine the validity of these devices in different types of physical activities under real-world conditions.

## Introduction

Since the turn of the 21st century, physical inactivity has increasingly become a global public health issue among youth [1]. In 2010, 81% of adolescents aged 11 to 17 years worldwide failed to achieve the World Health Organization–recommended amounts of moderate to vigorous physical activity (60 minutes or more per day). Of this proportion, girls were less active than boys (87% vs 78%, respectively) [2,3]. Similarly, nearly 70% of adolescents are categorized as insufficiently active, with girls having a higher prevalence of insufficient activity than boys (72% vs 68%, respectively) in China [4-6]. This is a serious issue [7] as physical inactivity in adolescence is associated with adult inactivity [8].

Physical inactivity is one of the leading risk factors for mortality, adding to the burden of noncommunicable diseases (NCDs) and affecting general health worldwide [9]. Physical inactivity among adolescents is significantly associated with many major health conditions, such as obesity, diabetes, and cardiovascular disease [10]. Young adults who are physically inactive during adolescence are also more likely to be overweight or obese than are their physically active counterparts [11].

Several behavior change methods exist to encourage youth to become more physically active. Self-monitoring of physical activity (PA) with activity devices is helpful to improve the PA levels of adolescents [12]. However, knowing the accuracy of activity devices in adolescents is necessary to identify current levels of PA and assess the effectiveness of intervention programs designed to increase PA.

Rapid advancement in the science and technology of wrist-wearable activity devices offers considerable potential for clinical applications, which may serve as cost-effective and attractive intervention methods for PA improvement apps. It is ideal to measure an adolescent’s PA during their usual living conditions to assess when and how long they are active and inactive in a typical day. In this context, measuring PA over the 24-hour day should not be limited to specific activities that can be measured in a laboratory; instead, it should be measured during free-living conditions [13]. Free-living conditions are different from laboratory settings as they offer a wider array of activities and situations for activity devices to measure PA. Accordingly, free-living validity information is important for researchers, fitness coaches, and consumers to choose the most appropriate activity device for their needs [14].

A few studies have examined the accuracy of wrist-wearable activity devices under free-living conditions [14-18]. Dominick [15] and Reid et al [14] reported that compared with the GT3X+ (ActiGraph LLC), the Flex (Fitbit Inc) can estimate total step counts accurately in the free-living conditions, but Chu [16] and Sushames et al [17] showed that the Flex overestimated total step counts with error rates of 15.5% to 47.2%. Other researchers have determined the validity of wrist-, waist-, and arm-wearable devices to monitor the total energy expenditure (TEE) under free-living conditions [18-20]. Brooke et al [19] found that TEE estimated by the Flex, FuelBand (Nike Inc), and Charge HR (Fitbit Inc) were similar to TEE obtained from the arm-worn SenseWear (BodyMedia) and Armband Mini (BodyMedia), but the Shine 2 (Misfit), UP3 (Jawbone), and Vivofit (Garmin Ltd) overestimated TEE with error rates of 15.2%, 22.8%, and 24.5%, respectively [19]. In addition, Dannecker et al [18] found that Fitbit devices significantly underestimated EE with an error rate of 28%. And Ferguson et al [20] found significant differences in TEE obtained from the Shine, UP, and Pulse O2 (Withings) compared with the SenseWear. To date, it appears that no studies have evaluated the accuracy of total step counts and TEE for a large number of wrist-worn activity devices simultaneously. Further, few studies have examined the accuracy of the devices for estimating physical activities in a metabolic chamber that can simulate free-living conditions and estimate energy expenditure of physical activity and TEE, especially in adolescents [14-17,19,20].

Considering this limited evidence, additional research is needed to determine the validity of wrist-wearable activity devices over long periods of time in controlled free-living conditions for adolescents. Hence, the study aimed to determine the validity of 11 wrist-wearable activity devices to monitor total step counts and TEE in adolescents under the stimulated free-living conditions.

## Methods

### Participants

Nineteen (10 male and 9 female) inactive and healthy participants aged 14 to 18 years volunteered to participate in the study. Participants were recruited from middle schools and community settings located within a 50 kilometer area of Shanghai University of Sport through online advertising, leaflets, and word of mouth. Inclusion criteria included free of metabolic disorders affecting energy expenditure and conditions that influence the ability to perform daily PA, a BMI from 18.5 kg/m^2^ to 23.9 kg/m^2^, and no attempt to lose weight within the past 2 years. Exclusion criteria included individuals with cardiovascular disease or musculoskeletal injury within the past 6 months and with acute illness, unstable chronic conditions, neurological disorders, and cognitive disorders. Each participant provided written informed consent, and all procedures were approved by the ethical committee of Shanghai University of Sport. The data were collected from December 2017 to June 2018.

### Procedures

Participants completed 2 study visits. We asked the participants to refrain from vigorous physical activities on the day before each experiment. At the first visit, participants gave informed consent, had their weight, percent body fat, height, and maximum oxygen uptake (VO_2_max) measured while in a fasting state (12 hours postprandial). Participants also completed the long form of the International Physical Activity Questionnaire [21] to determine information about their lifestyle habits. Each participant’s energy intake in the metabolic chamber was calculated by multiplying the basal metabolic rate (BMR) predicted by using revised Harris-Benedict equation by 1.55, which was the PA level assumed for a standardized day.

At the second visit, each participant was given 12 wrist-wearable activity devices to wear for 24 hours in the metabolic chamber. We selected these devices based on domestic and foreign sales rankings and the attention of the interrelated research field. Nine were worn on their nondominant wrist in a random order (GT3X+, Flex, Vivofit, B2 [Huawei Technologies Co Ltd], UP3 [Jawbone], Shine 2 [Misfit], Loop [Polar Electro], Pulse O2 [Withings], Mi Band 2 [XiaoMi], and three were worn on their dominant wrist in random order (Amazfit [Huami Corp], Bong 2s [Meizu], GOLiFE Care-X [GoYourLife Inc]). Characteristics of the activity devices are described in Table 1.

**Table 1:** Activity devices details, set up parameters and analysis software.

Device	Retail price ($)	Steps	Distance	Energy expenditure			Sleep time			Active time		Wear site		Setup parameters	Software	
				Basal	Activity	Deep		Light							Setup	Analysis
GT3X+ (ActiGraph)	249.00	x^a^	—^b^	—	x	—		—	x		hip, wrist		H^c^, W^d^, sex, DOB^e^, 30 Hz, 60 s epoch		Actilife V6.0	Actilife V6.0
Amazfit (Huami)	43.46	x	—	x	x	x		x	—		wrist		H, W, sex, DOB		Midong iPad app	Midong iOS app
Bong 2s (Meizu)	18.75	x	—	—	x	x		x	x		wrist		H, W, sex, DOB		Bong iPad app	Bong iOS app
Flex (Fitbit)	130.52	x	x	+^f^	+	—		—	x		wrist		H, W, sex, DOB		Fitbit iPad app	Fitbit iOS app
Vivofit (Garmin)	72.53	x	x	x	x	x		x	—		wrist		H, W, sex, DOB		Connect iPad app	Connect iOS app
GOLiFE Care-X (GoYourLife)	28.78	x	x	+	+	x		x	—		wrist		H, W, sex, DOB		GOLiFE Fit iPad app	GOLiFE Fit iOS app
B2 (Huawei)	116.13	x	x	—	x	x		x	x		wrist		H, W, sex, DOB		Huawei wearable iPad app	Huawei wearable iOS app
UP3 (Jawbone)	159.74	x	x	x	x	x		x	x		wrist		H, W, sex, DOB		UP iPad app	UP iOS app
Shine 2 (Misfit)	116.13	x	x	+	+	x		x	x		wrist		H, W, sex, DOB		Misfit iPad app	Misfit iOS app
Loop (Polar Electro)	142.44	x	x	+	+	x		x	x		wrist		H, W, sex, DOB		Polar iPad app	Polar iOS app
Pulse O2 (Withings)	137.8	x	x	+	+	x		x	—		wrist		H, W, sex, DOB		Withings iPad app	Withings iOS app
Mi Band 2^g^ (XiaoMi)	21.66	x	x	—	x	x		x	x		wrist		H, W, sex, DOB		Xiaomi Sport iPad app	Xiaomi Sport iOS app

^a^x: feature present.

^b^—: feature absent.

^c^H: height.

^d^W: weight.

^e^DOB: date of birth.

^f^+: sum of basal and activity energy expenditures.

^g^Device no longer on the market.

Each participant stayed in the metabolic chamber alone for 24 hours to measure TEE in a simulated free-living environment. Moreover, the researchers would remind the participants to perform daily physical activities (eg, watching TV, sleeping, eating lunch) according to the schedule of activities. The schedule of activities performed in the metabolic chamber is shown in Table 2. Since daily PAs are performed frequently for short durations in actual life, each activity was limited to a period of 30 minutes, except for doing housework and radio gymnastics, which were 10 minutes long.

**Table 2:** Schedule of activities during the metabolic chamber stay.

Timetable	Activity
19:40	Enter chamber
20:00-22:00	Watch TV
22:00-22:45	Measure RMR^a^
22:45-23:00	Prepare to sleep
23:00-07:00	Sleep
07:00-07:15	Prepare to measure BMR^b^
07:15-08:00	Measure BMR
08:00-08:15	Eat breakfast
08:15-08:45	Listen to music
08:45-09:15	Read
09:15-10:00	Watch videos
10:00-10:10	Do housework
10:10-10:20	Do video calisthenics
10:20-10:50	Slow walk at the speed of 3.2 km/h
10:50-11:20	Play on the phone
11:20-11:50	Type
11:50-12:05	Eat lunch
12:05-13:00	Midday sleep
13:00-13:30	Read
13:30-14:00	Type
14:00-14:30	Fast walk at the speed of 5.6 km/h
14:30-15:00	Listen to music
15:00-25:30	Read
15:30-16:00	Type
16:00-16:30	Run at the speed of 8 km/h
16:30-17:15	Watch videos
17:15-17:45	Play on the phone
17:45-18:00	Eat dinner
18:00-18:30	Listen to music
18:30-19:00	Write
19:00-19:30	Slow walk at a self-selected speed
19:30-20:00	Watch TV
20:20	Leave chamber

^a^RMR: resting metabolic rate.

^b^BMR: basal metabolic rate.

### Materials and Measures

#### Demographics, Anthropometrics, and Cardiorespiratory Fitness

A digital scale (Takei Kiki Kogyo Co Ltd) was used to measure body weight to the nearest 0.1 kg while participants were dressed in light clothing. Height was measured to the nearest 0.1 cm by using an electronic stadiometer with participants standing barefoot. BMI was computed as kg/ m^2^. Percent body fat was measured by dual-energy x-ray absorptiometry (Lunar Prodigy, GE Healthcare).

#### Total Energy Expenditure

The TEE was measured using a whole metabolic chamber (3.85 m width × 2.85 m depth × 2.5 m height; FHC-20S, Fuji Medical Science Co Ltd), which contains a toilet, wash stand, bed, desk with chair, and treadmill. Participants can sleep, eat, and do different physical activities in the chamber. The temperature and relative humidity of incoming fresh air were maintained at 25.0°C (±0.5°C) and 50.0% (±3.0%), respectively. The sample air is dehumidified using a gas-sampling unit (SCC-C, ABB Corp) and analyzed using a mass spectrometer (Prima PRO, Thermo Fisher Scientific) [22]. The accuracy of VO^2^ and VCO^2^ measured by metabolic chamber is 99.8% to 99.9%. Once a month, the accuracy and precision of the respiratory chamber are assessed by 24-hour propane combustion tests. The chamber software allows the measurement of energy expenditure with high-time resolution by detecting changes in activity level [23].

#### Step Counts

The GT3X+ is the most widely used accelerometer to monitor physical activity. The data are displayed as counts, which represents movement intensity and step counts taken. Lee et al [24] reported that the GT3X+ counted 98.5% of the steps compared with the Yamax Digiwalker SW-701 (Yamasa Tokei Keiki Co Ltd) pedometer during free living. We used the wrist-worn GT3X+ to monitor PA while participants were in the metabolic chamber.

### Data Processing

Before data collection, the devices were set up with unique user accounts using the parameters of weight, height, gender, and date of birth. Data from the devices were recorded at the beginning and end of each session. Data were downloaded from each device-specific app and uploaded to an iPad (Apple Corp). Step counts from the GT3X+ were downloaded and analyzed using ActiLife 6 software. The Mi Band 2, B2, and Bong 2s yielded estimates of activity EE without accounting for the resting metabolic rate according to the manufacturer’s instructions. To facilitate direct comparisons, we calculated the resting energy expenditure for each participant using the following revised Harris-Benedict equation [25]:

- *Male*=88.362+[13.397*weight(*kg*)]+[4.799*height (*cm*)]–(5.677*age)
- *Female*=447.593+[9.247*weight(*kg*)]+[3.098*height (*cm*)]–(4.330*age)

Estimated resting EE values were added to the measured activity EE values from the activity devices to calculate the total EE.

### Statistical Analysis

Paired *t* tests were the statistical model adopted for the sample size calculation. The medium ES=0.5 was determined based on the variable of step in the study by Dominick et al [15] (Cohen *d*=0.4). Therefore, we estimated that 17 paired observations would be needed to achieve 80% power to detect the primary outcome variables between the reference devices and activity devices, with 2-sided alpha=.05. To allow for potential withdrawals, 19 participants were randomized.

We analyzed all data using SPSS Statistics version 19.0 (IBM Corp). Data were first checked for normality using standardized skewness and kurtosis values. The results showed that the data in this study were normally distributed. The mean and standard deviation were presented for normally distributed data. Paired *t* tests for normally distributed data were used to analyze differences between the activity devices and the criterion measures: total EE from the metabolic chamber (mcTEE) and step counts from the GT3X+ (AGsteps). A significance level of .05 was used to guide statistical decisions.

Pearson correlation analyses were used to determine the association between the summary scores from each device and the criterion measures. Mean bias (estimated values – measured values) was computed to show the overall underestimation or overestimation of TEE and total step counts by each device compared with the criterion measures at the group level. Mean absolute percentage error (MAPE, [estimated values – measured values] / measured values × 100%) was calculated to quantify the differences between the wrist-wearable activity devices and the criterion measures at the individual level. MAPE accounts for each individual participant’s error while avoiding cancellation of errors from underestimation and overestimation [26]. Bland-Altman statistics were performed to determine the 95% limits of agreement to further evaluate individual variations in a more systematic way for each device compared with the criterion measures.

Paired *t* tests are designed to test for differences rather equivalence. The failure to reject the null hypothesis of no difference simply cannot be used to infer agreement or equivalence. Therefore, equivalence testing is used to statistically examine measurement agreements between devices and criterion measures at the group level [26]. Since there are no definitive guidelines to follow to determine the accuracy of the equivalence tests, we selected a 10% error zone. The devices are considered to be equivalent to the criterion measure (with 95% precision) if the 90% confidence interval for a mean of estimated values falls into the defined equivalence zone [27].

## Results

Nineteen participants met the eligibility criteria, agreed to participate, and completed the study. Participants’ ages ranged from 14 to 18 (mean 17.3 [SD 1.3]) years. BMI ranged from 17.8 to 24.4 (mean 20.5 [SD 1.8]) kg/m^2^, and percent body fat ranged from 6.1% to 36.8% (mean 24.0% [SD 9.7%]). The information from the long form of the International Physical Activity Questionnaire confirmed that participants were physically inactive (mean moderate to vigorous PA 95-150 minutes per week). All participants were right hand dominant.

The Pearson correlation coefficient between the wrist-wearable activity devices and the criterion measures for TEE and step counts are displayed in Table 3. All wrist-wearable activity devices were strongly correlated with mcTEE with correlations ranging from *r*=.78 (Shine 2, Amazfit; *P*<.001) to *r*=.96 (Loop; *P*<.001) for TEE. Only the Flex and Vivofit were significantly correlated with AGsteps with *r*=.54 and *r*=.57, respectively (*P*<.05).

**Table 3:** The Pearson correlation coefficient between wrist-wearable activity devices and criterion measures for total energy expenditure (kcal) and step counts.

Device	McTEE^a^	*P* value	AGsteps^b^	*P* value
Amazfit (Huami)	0.78	<.001	0.45	.06
Bong 2s (Meizu)	0.85	<.001	0.44	.07
Flex (Fitbit)	0.92	<.001	0.54	.02
Vivofit (Garmin)	0.85	<.001	0.57	.01
GOLiFE (GoYourLife)	0.88	<.001	0.20	.42
B2 (Huawei)	0.87	<.001	0.40	.10
UP3 (Jawbone)	0.87	<.001	0.46	.05
Shine 2 (Misfit)	0.78	<.001	0.26	.29
Loop (Polar Electro)	0.96	<.001	0.44	.07
Pulse O2 (Withings)	0.86	<.001	0.22	.38
Mi Band 2 (XiaoMi)	0.91	<.001	0.42	.09

^a^McTEE: total energy expenditure from the metabolic chamber.

^b^AGsteps: total steps from the GT3X+ (ActiGraph).

The mean, standard deviation, and bias between wrist-wearable activity devices and the criterion measures are displayed in Table 4. For TEE, there were no significant differences between the Mi Band 2, UP3, Vivofit, and Bong 2s with mcTEE (*P*>.05). The Flex, Shine 2, and Loop overestimated TEE significantly as noted by the positive bias values ranging from 7.0% (Loop) to 19.0% (Shine 2; *P*<.05). On the contrary, Amazfit, GOLiFE, B2, and Pulse O2 underestimated TEE significantly as noted by the negative bias values ranging from 5.6% (GOLiFE) to 26.6% (Amazfit; *P*<.05). For step counts, there were no significant differences between the Bong 2s, GOLiFE, and Pulse O2 with AGsteps (*P*>.05). The remaining devices overestimated the AGsteps significantly as noted by the positive bias values ranging from 9.7% (Shine 2) to 24.3% (Loop; *P*<.05).

**Table 4:** Mean, standard deviation, and bias between wrist-wearable activity devices and criterion measures for total energy expenditure (kcal) and step counts (n=19)a.

Device	TEE_P_ (kcal)^b^	Bias	*P* value	Step count	Bias	*P* value
Amazfit	1496.6 (249.1)	–542.0 (188.2)	<.001	11,910.3 (1864.3)	1766.6 (1753.8)	<.001
Bong 2s^c^	2037.7 (208.8)	–0.9 (164.5)	.98	9586.4 (1600.6)	–557.4 (1602.6)	.16
Flex	2325.6 (272.2)	287.0 (118.7)	<.001	12,228.2 (1377.3)	2084.0 (1327.0)	<.001
Vivofit	2040.5 (290.4)	1.9 (162.2)	.96	12,411.7 (1396.8)	2267.9 (1300.6)	<.001
GOLiFE	1925.0 (246.8)	–113.6 (144.9)	<.001	11,111.8 (2374.5)	968.1 (2497.2)	.12
B2^b^	1922.8 (258.1)	–115.9 (146.7)	<.001	12,193.9 (1246.1)	2050.1 (1456.7)	<.001
UP3	1970.5 (282.4)	–68.1 (148.5)	.06	12,031.5 (1430.4)	1887.7 (1464.4)	<.001
Shine 2	2426.5 (324.4)	387.9 (209.7)	<.001	11,127.2 (1590.4)	983.4 (1820.4)	.04
Loop	2181.8 (312.1)	143.2 (92.4)	<.001	12,613.0 (1785.6)	2469.2 (1714.2)	<.001
Pulse O2	1886.0 (261.4)	–152.6 (154.2)	<.001	11,107.1 (1984.9)	963.3 (2160.9)	.08
Mi Band 2^c^	1979.1 (239.0)	–59.5 (128.5)	.06	11,986.3 (1487.9)	1842.6 (1560.2)	<.001

^a^ Criterion values: McTEE 2038.6 (299.8) kcal; AGsteps 10143.8 (1396.5).

^b^TEE_P_: predicted total energy expenditure.

**^c^**Add rest energy expenditure.

**Figure 1:**
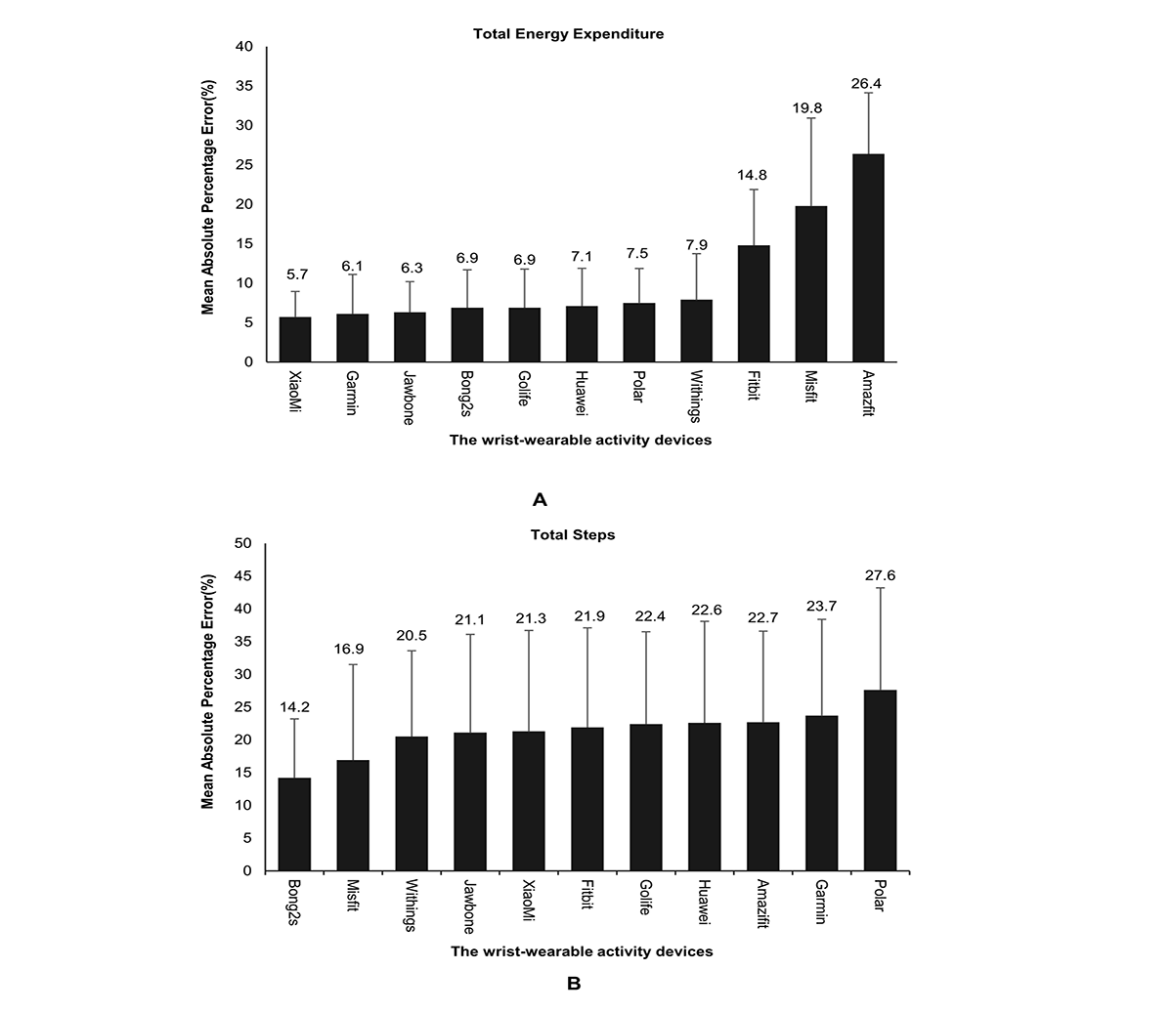
Mean absolute percentage error for total energy expenditure and steps estimated by wrist-wearable activity devices.

MAPEs for the various devices are illustrated in Figure 1. For TEE, the magnitude of MAPE was least for the Mi Band 2 (5.7%) and highest for the Amazfit (26.4%; Figure 1A). For step counts, the magnitude of MAPE was least for the Bong 2s (14.2%) and highest for the Loop (27.6%; Figure 1B).

**Figure 2:**
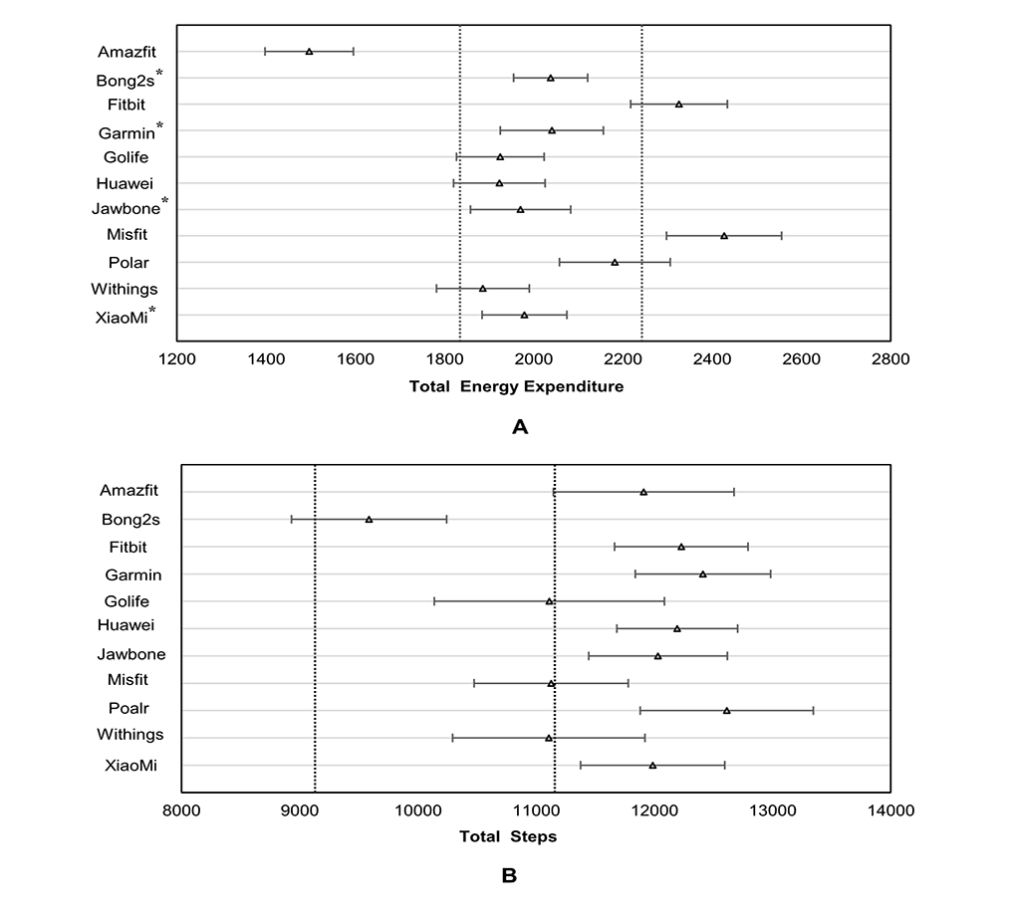
Agreement on total energy expenditure (kcal) and step counts between criterion measured and devices on 95% equivalence testing. Dashed lines indicate the equivalence zone from criterion measured. Dark lines indicate the 90% confidence interval of estimated values from the devices. *Within the equivalence zone. ∆: mean value estimated by activity devices.

Equivalence test results are displayed in Figure 2. For TEE, the calculated 90% confidence interval from the Mi Band 2, UP3, Vivofit, and Bong 2s fell within the equivalence zone, indicating equivalence with mcTEE at the group level. The B2 and GOLiFE were close to the equivalence zone (Figure 2A). For step counts, no device was equivalent with AGsteps, however the Bong 2s was closest to the equivalence zone (Figure 2B). All the Bland-Altman scatter plots displayed no systematic bias for all wrist-wearable activity devices (Multimedia Appendix 1).

## Discussion

### Principal Findings

This study aimed to determine the validity of 11 wrist-wearable activity devices for monitoring TEE and total step counts in adolescents during simulated free-living conditions. For TEE, we found that the predicted values by all wrist-wearable activity devices were strongly correlated with TEE obtained from the metabolic chamber and the Mi Band 2, UP3, Vivofit, and Bong 2s measured TEE accurately. For step counts, only the Flex and Vivofit had moderate correlations with the steps obtained by the GT3X+. The Bong 2s, GOLiFE, and Pulse O2 steps were similar to AGsteps. Overall, the wrist-activity devices listed above tended to show good validity when monitoring TEE but not in monitoring step counts at the individual and group levels.

For TEE, the UP3 and Pulse O2 underestimated TEE, and the Flex and Shine 2 overestimated TEE. This finding aligns with previous studies [28,29] showing the UP3, Shine 2, FuelBand, and Pulse O2 compared with criterion measures such as the SenseWear and TEE from a metabolic chamber. The MAPE for the Pulse O2 and Shine 2 (10% to 20%) were similar to those obtained by Ferguson [20]. However, for the UP3, the MAPE in this study was 6.3% which differs widely from values observed by Ferguson [20] and Brooke [19] that reported error rates of more than 29.8% and 22.8%, respectively. Murakami showed the UP3 had a MAPE of 13% compared with TEE from the metabolic chamber and an error rate of more than 20% compared with doubly labeled water. However, Murakami [28] reported using a different reference standard for TEE obtained from the metabolic chamber than the one used in this study. It should be noted, however, that the metabolic chamber had higher accuracy and precision for total daily energy expenditure than doubly labeled water according to the study by Melanson et al [29]. The comparisons may have more accuracy when considering the metabolic chamber as the gold standard for measuring TEE.

This is the first study to examine the validity of Mi Band 2, B2, and Bong 2s on estimating TEE. All three devices were significantly correlated with the TEE, and the Mi Band 2 and Bong 2s estimated TEE accurately. Since the Mi Band 2, B2, and Bong 2s only provided PA energy expenditure output, the predicted resting metabolic rate using the revised Harris-Benedict equation was added to PA energy expenditure measured by these devices in order to provide a more appropriate comparison with TEE in our study. Accordingly, interpretation of the results for these devices requires caution.

This study found that all of the wrist-wearable activity devices overestimated the AGsteps with the exception of the Bong 2s. It is likely that recording total step counts in a free-living setting over a longer duration (ie, 24 hours) resulted in different findings from studies that measured walking for shorter periods of time [30-32]. However, there are similarities in results with Rosenberger et al [13], who showed the UP3 overestimated total steps on the order of 20%, and by Chu et al [16] and Sushames et al [17], who showed that the Flex overestimated total steps from 15.5% to 47.2% (both *P*<.05). Unlike our study, Dominick et al [15] and Reid et al [14] showed the Flex can monitor total steps accurately. However, this discrepancy may be due to different characteristics of the participants studied. The proportion of female participants was nearly 80% in the previous two studies [14,15]. Ferguson et al [20] and Farina et al [33] found that the UP3 and Shine 2 underestimated total steps by 3% and 11%, respectively. This differed from our study, which showed the UP3 and Shine 2 overestimated total step counts by 16.9% and 21.1%, respectively. A possible reason for the underestimation observed by Ferguson [20] and Farina [33] is that their participants were aged 20 to 84 years while the participants in our study were aged 14 to 18 years. In past studies, older adults were shown to be less active compared with younger people [7]. With the lower activity levels and shorter time for monitoring exercise duration, a relatively small range of movement may be overlooked by sensor [34-37]. Therefore, studies are needed with wrist-wearable activity devices in persons with wide age differences who are measured in similar experimental settings so as to assess the accuracy of wrist-wearable activity devices objectively and widely. Further, few [20,28] or no studies have assessed some of these devices, such as the Pulse O2, Mi Band 2, B2, Bong 2s, Amazfit, and GOLiFE Care-X, as done in this study.

As the criterion measure of step counts, the GT3X+ was worn on the nondominant wrist in this study in order to standardize the study design and minimize the measurement variation introduced by the placement of the devices. Compared with hip-worn accelerometers, wrist-worn accelerometers may be less intrusive, particularly during sleep, and may thus engender higher compliance. Wrist-worn accelerometers have been used to monitor children’s and adolescents’ physical activity for nearly two decades [38]. In their PA surveillance activities, the National Health and Nutrition Examination Survey previously used a uniaxial accelerometer worn on hip to assess PA (2003-2004 and 2005-2006) but has now changed its protocol, asking participants to wear a triaxial accelerometer on the wrist instead of hip in their 2011-2014 surveillance systems, which include persons aged 6 years and older [39].

As a whole, in this study all wrist-wearable activity devices overestimated step counts by 963 to 2469 steps as compared with the GT3X+. It is noteworthy that users may reduce PA if wrist-wearable activity devices overestimate steps, as this may cause the illusion of achieving the goal of fitness and prevent consumers achieving the goal indirectly. This specific type of information about the accuracy of step monitoring devices may be valuable to consumers considering purchasing such devices. That said, contemporary wrist-wearable activity devices have emphasized wrist locations by the manufacturers for their less obstructive placement and user’s convenience in checking their progress throughout the day. Wrist locations also facilitate integration with telecommunications features (ie, smart watch), enable sleep detection, and promote participant compliance [40].

In this study, we found that the price and performance of wrist-wearable activity devices seems to be unrelated. The most inexpensive wrist-wearable activity device, Bong 2s, was one of the best performing activity devices, while more expensive activity devices (Loop, Flex, B2) showed a large difference in accuracy, a finding similar to results in the study by Ferguson et al [20]. It is likely that the addition of smartphone connectivity, intelligence, wearability, and esthetics contribute to higher priced wrist-wearable activity devices.

### Strengths and Limitations

Our study has some strengths. First, participants were adolescents aged 14 to 18 years. In all previous studies, samples included adults and older people only. The addition of this study, combined with investigations with a broader age range of participants, can provide more confidence that the results can be generalized to a broader population, especially teenagers who typically have lower levels of physical activity in many societies [4]. Second, we used the metabolic chamber as a gold standard criterion measure for TEE. A high-precision metabolic chamber allowed precise measurement of EE which facilitated the output of credible results. Beyond that, the cubage of the metabolic chamber is 11.4 m^2^, similar to a household room. Accordingly, we could simulate a free-living environment to monitor daily behavior in a real-time 24-hour daily life. Third, compared with previous studies, we examined the accuracy of a wide range of wrist-wearable activity devices: Mi Band 2, Flex, UP3, Vivofit, Shine 2, B2, Bong 2s, GOLiFE Care-X, Pulse O2, Amazfit, and Loop. The price of the wrist-wearable activity devices ranged from US $18 to $250, which is suitable for people in different consumer stratums. Collectively, the results in our study can inform decision making about the use of wrist-wearable activity devices.

This study is not without limitations. First, we did not assess the reliability of the wrist-wearable activity devices. Poor reliability can negatively impact validity. In further studies, we need to test the reliability of wrist-wearable activity devices to ensure consistency among the different brands. Second, we need to further test wrist-wearable activity device monitors to assess multiple parameters such as different types of PA EE, distance, time of various intensity, sleep, and so on, which may impact the validity of the devices. Additionally, the results of our research should be carefully considered for application to overweight and obese people. Finally, according to the time schedule in the metabolic chamber, there were many activities of daily life (eg, listening to music, doing housework, writing), but these data were not revealed in detail in this paper.

### Conclusions

In conclusion, the Mi Band 2, UP3, Vivofit, and Bong 2s wrist-worn activity devices estimated TEE accurately both at individual and group level as compared to the TEE obtained in a metabolic chamber. The Bong 2s, GOLiFE, and Pulse O2 were similar to total step counts recorded by the GT3X+ at the individual level. No devices were equivalent with total step counts from the GT3X+ at the group level. With the upgrade and expansion of the measurement abilities of the wrist-wearable activity devices, the research field should regularly assess the accuracy of new devices to ensure that the wrist-wearable activity devices can be used with confidence in scientific research and by practitioners in daily life.

## Appendix

Multimedia Appendix 1 Bland-Altman scatterplots of steps and total energy expenditure for all wrist-wearable activity devices.

## Abbreviations

AGsteps: step counts from the ActiGraph GT3X+
BMR: basal metabolic rate
MAPE: mean absolute percentage error
mcTEE: total energy expenditure from metabolic chamber
NCD: noncommunicable disease
PA: physical activity
TEE: total energy expenditure
