# Supplementary material for: Validity of Wrist-Wearable Activity Devices for Estimating Physical Activity in Adolescents: Comparative Study
Source: JMIR Mhealth Uhealth. 2021 Jan 7;9(1):e18320. doi: 10.2196/18320 (PMC7819784; doi:10.2196/18320)
Supplement: Multimedia Appendix 1 [file mhealth_v9i1e18320_app1.pdf]

**Steps:**

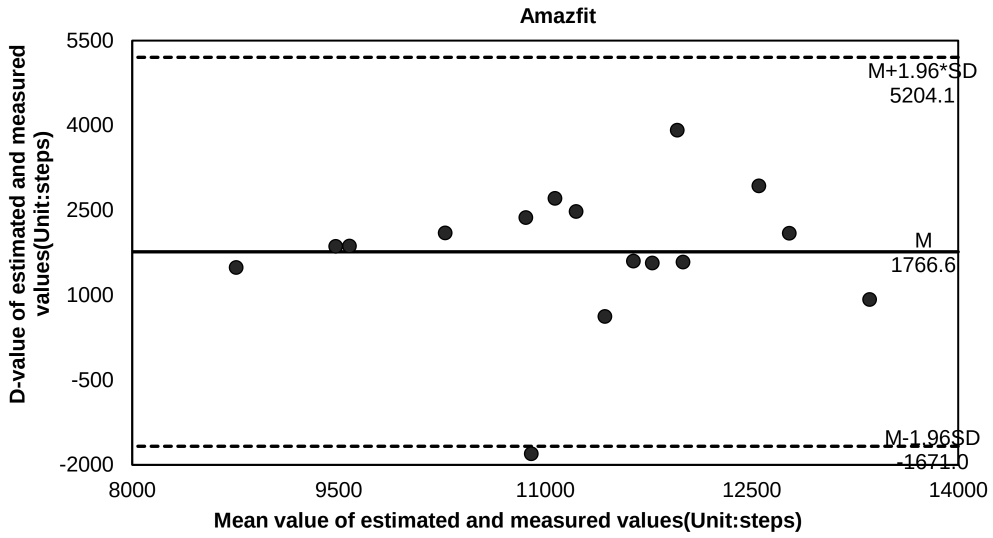

Amazfit
5500
4000
2500
1000
-500
-2000
8000
9500
11000
12500
14000
M+1.96*SD
5204.1
M
1766.6
M-1.96SD
-1671.0
D-value of estimated and measured values(Unit:steps)
Mean value of estimated and measured values(Unit:steps)


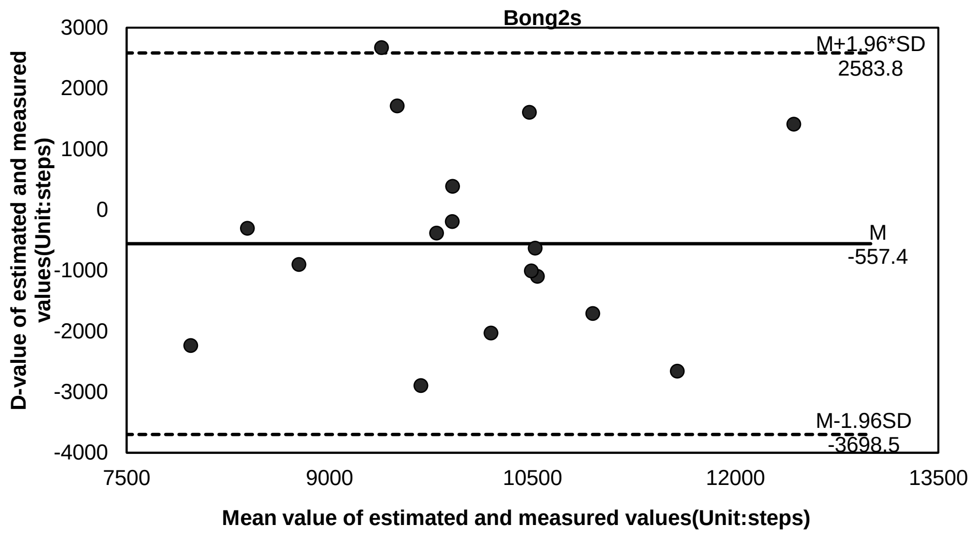

Bong2s
3000
2000
1000
0
-1000
-2000
-3000
-4000
7500
9000
10500
12000
13500
M+1.96*SD
2583.8
M
-557.4
M-1.96SD
-3698.5
D-value of estimated and measured values(Unit:steps)
Mean value of estimated and measured values(Unit:steps)


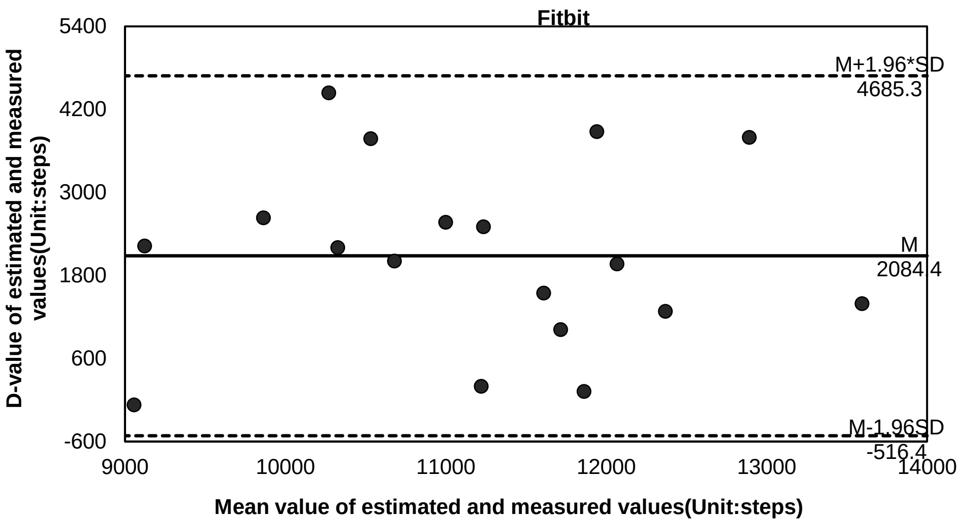

Fitbit
5400
4200
3000
1800
600
-600
9000
10000
11000
12000
13000
14000
M+1.96*SD
4685.3
M
2084.4
M-1.96SD
-516.4
D-value of estimated and measured values(Unit:steps)
Mean value of estimated and measured values(Unit:steps)

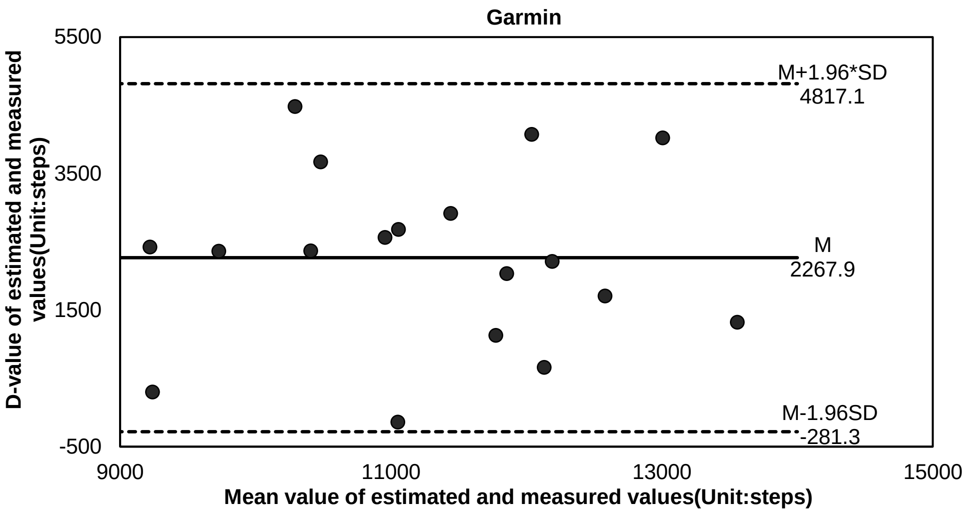
Garmin
5500
3500
1500
-500
M+1.96*SD
4817.1
M
2267.9
M-1.96SD
-281.3
9000
11000
13000
15000
D-value of estimated and measured values(Unit:steps)
Mean value of estimated and measured values(Unit:steps)

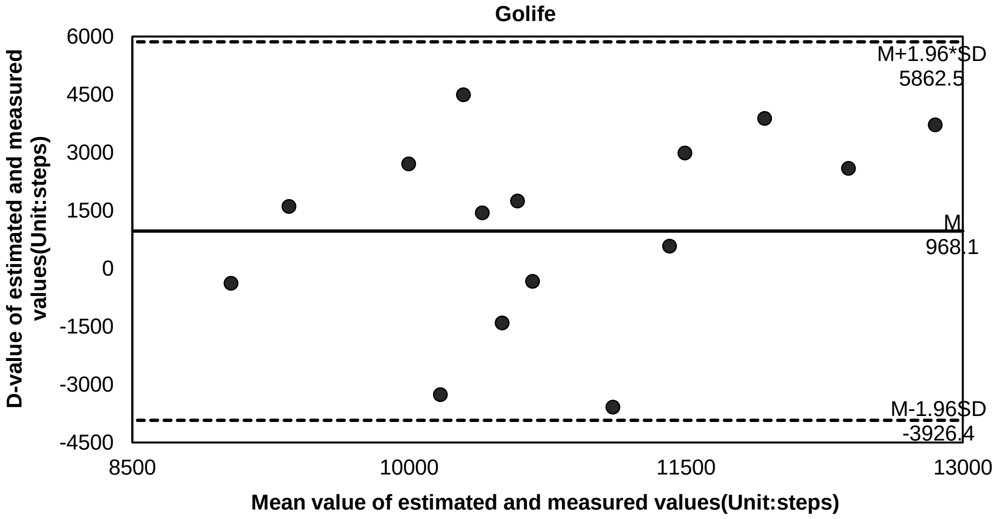
Golife
6000
4500
3000
1500
0
-1500
-3000
-4500
M+1.96*SD
5862.5
M
968.1
M-1.96SD
-3926.4
8500
10000
11500
13000
D-value of estimated and measured values(Unit:steps)
Mean value of estimated and measured values(Unit:steps)

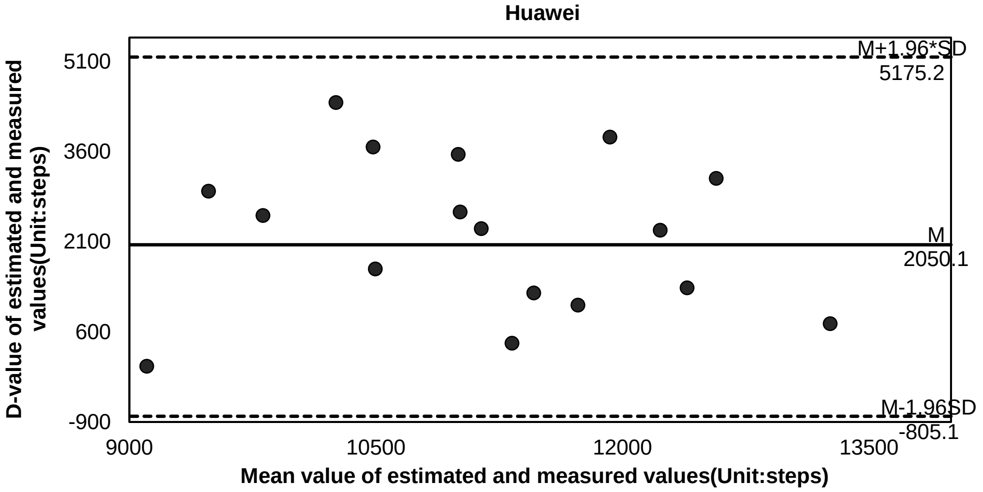
Huawei
5100
3600
2100
600
-900
M+1.96*SD
5175.2
M
2050.1
M-1.96SD
-805.1
9000
10500
12000
13500
D-value of estimated and measured values(Unit:steps)
Mean value of estimated and measured values(Unit:steps)

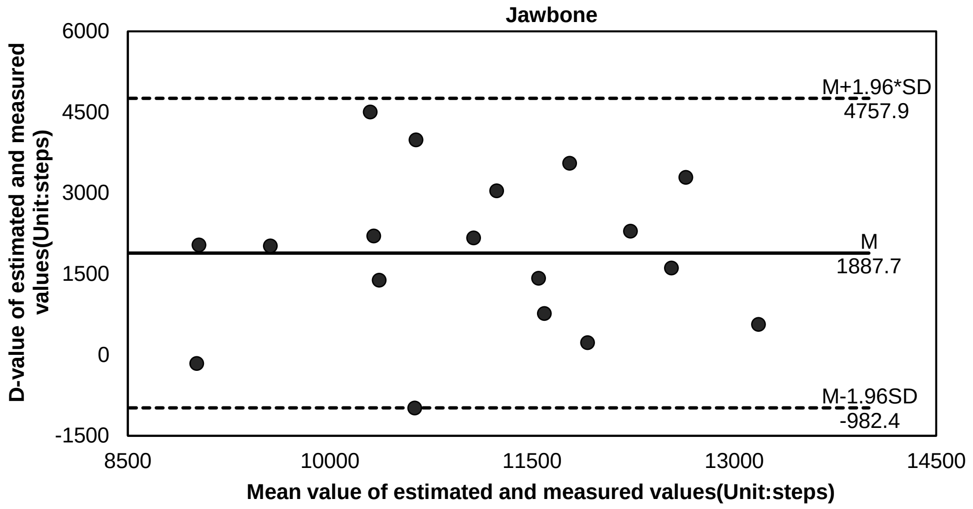

Jawbone
M+1.96*SD
4757.9
M
1887.7
M-1.96SD
-982.4
6000
4500
3000
1500
0
-1500
8500
10000
11500
13000
14500
D-value of estimated and measured values(Unit:steps)
Mean value of estimated and measured values(Unit:steps)


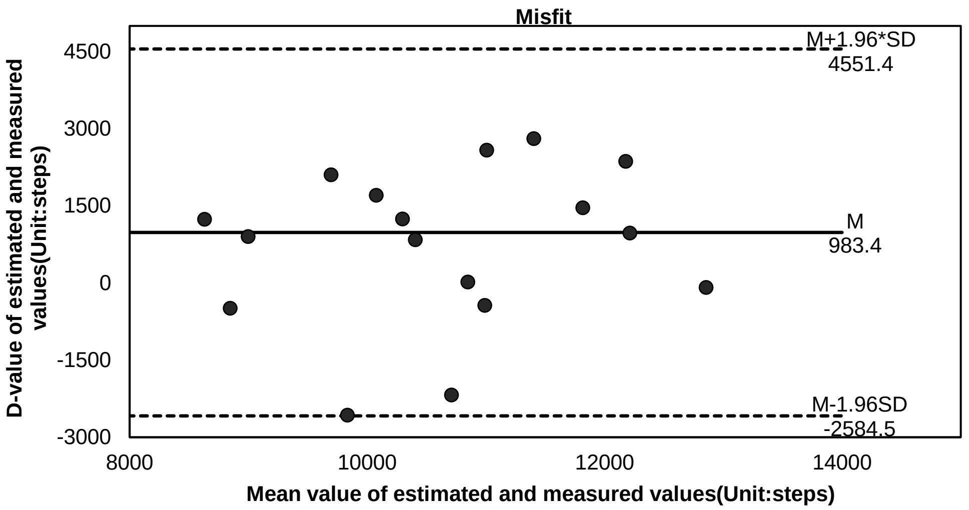

Misfit
M+1.96*SD
4551.4
M
983.4
M-1.96SD
-2584.5
4500
3000
1500
0
-1500
-3000
8000
10000
12000
14000
D-value of estimated and measured values(Unit:steps)
Mean value of estimated and measured values(Unit:steps)


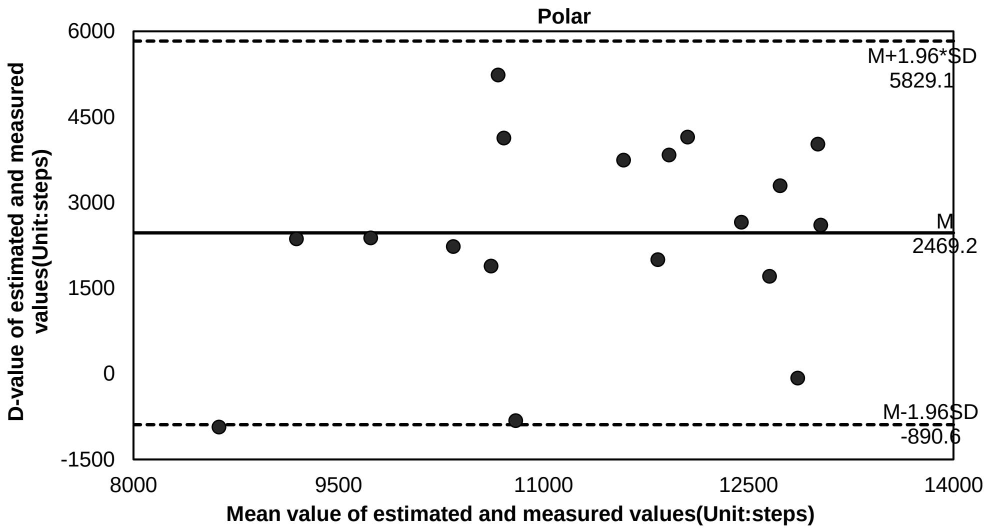

Polar
M+1.96*SD
5829.1
M
2469.2
M-1.96SD
-890.6
6000
4500
3000
1500
0
-1500
8000
9500
11000
12500
14000
D-value of estimated and measured values(Unit:steps)
Mean value of estimated and measured values(Unit:steps)

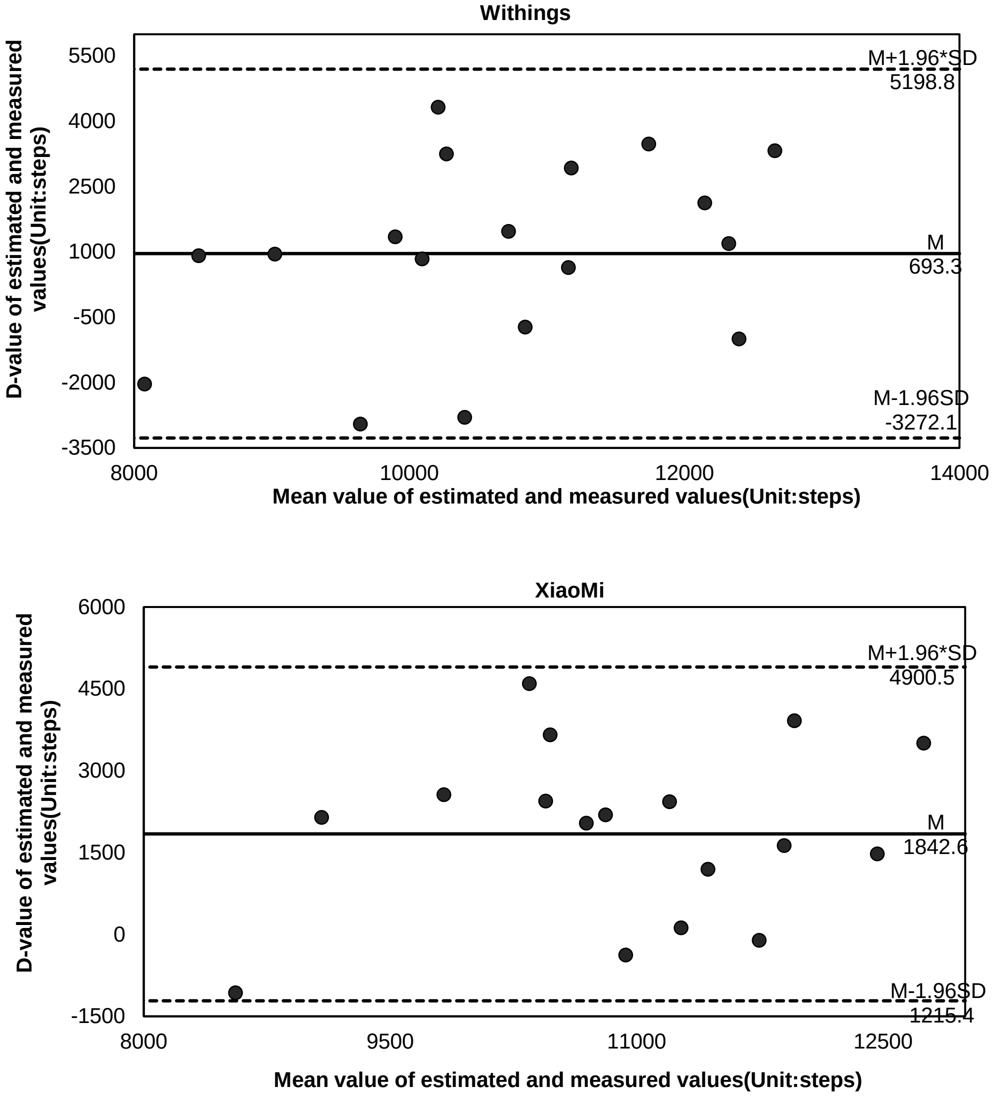
Withings
D-value of estimated and measured values(Unit:steps)
5500
4000
2500
1000
-500
-2000
-3500
M+1.96*SD
5198.8
M
693.3
M-1.96SD
-3272.1
8000
10000
12000
14000
Mean value of estimated and measured values(Unit:steps)
XiaoMi
D-value of estimated and measured values(Unit:steps)
6000
4500
3000
1500
0
-1500
M+1.96*SD
4900.5
M
1842.6
M-1.96SD
1215.4
8000
9500
11000
12500
Mean value of estimated and measured values(Unit:steps)

**a**

**Energy expenditure:**

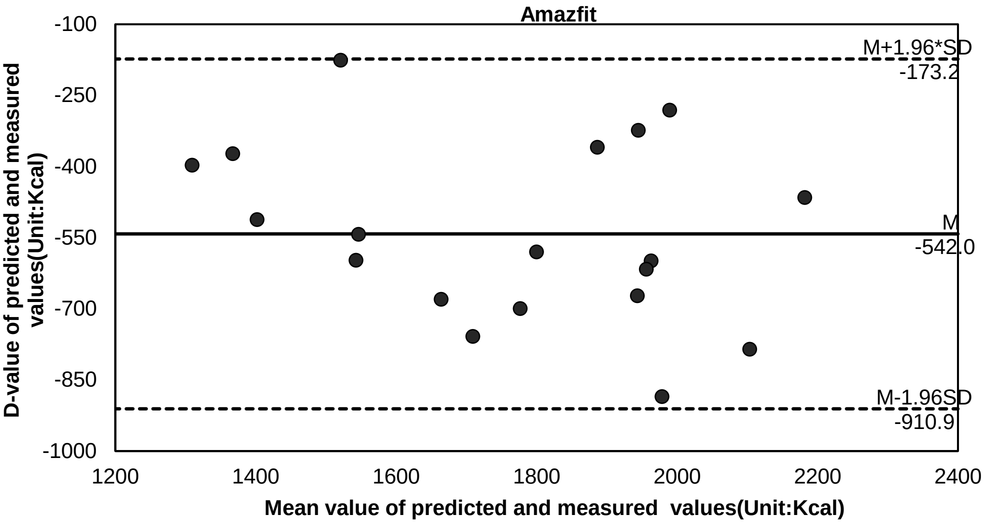


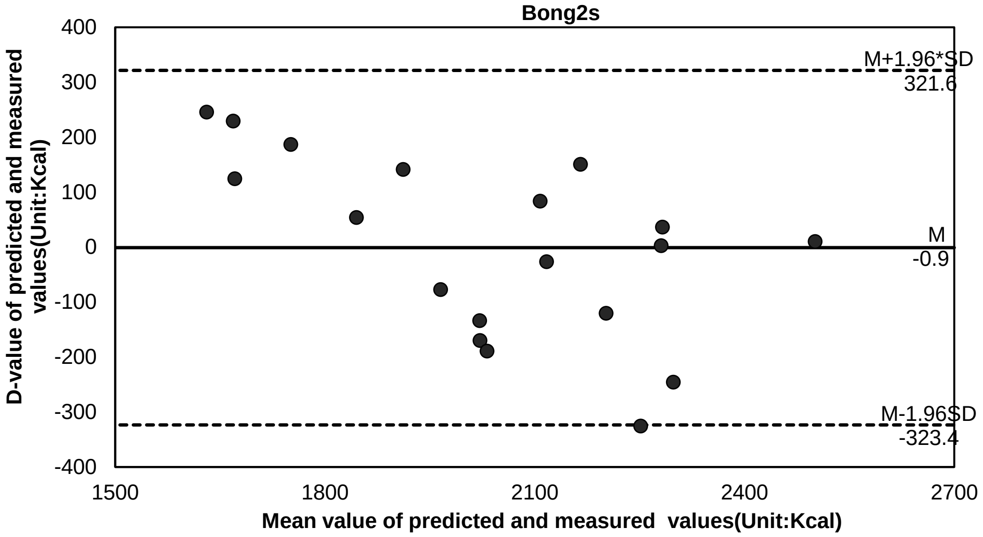


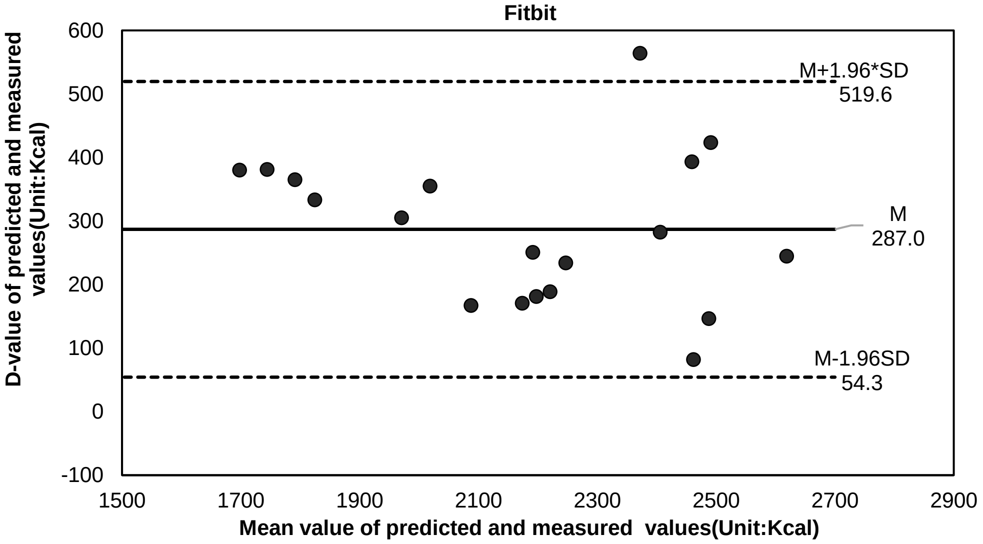

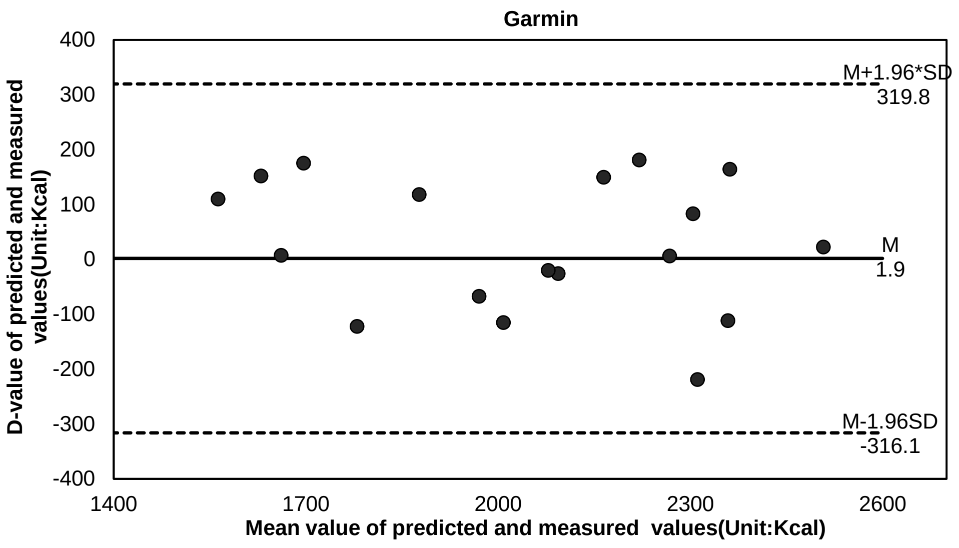
Garmin
400
300
200
100
0
-100
-200
-300
-400
1400
1700
2000
2300
2600
M+1.96*SD
319.8
M
1.9
M-1.96SD
-316.1
D-value of predicted and measured values(Unit:Kcal)
Mean value of predicted and measured values(Unit:Kcal)

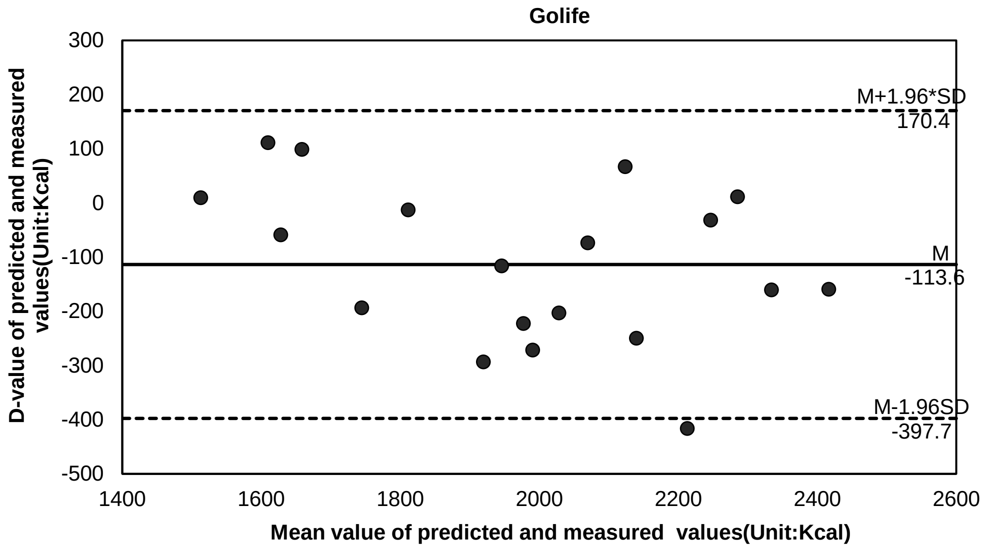
Golife
300
200
100
0
-100
-200
-300
-400
-500
1400
1600
1800
2000
2200
2400
2600
M+1.96*SD
170.4
M
-113.6
M-1.96SD
-397.7
D-value of predicted and measured values(Unit:Kcal)
Mean value of predicted and measured values(Unit:Kcal)

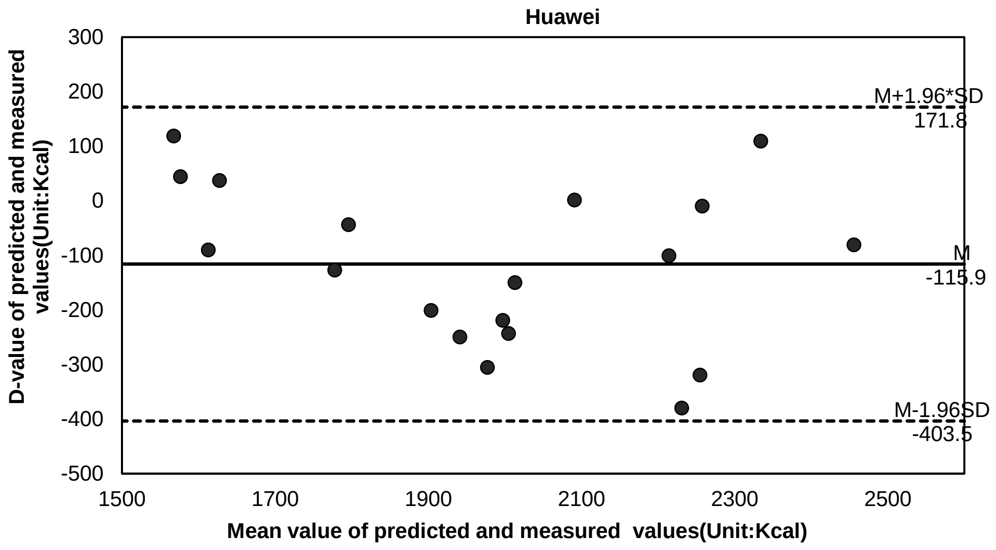
Huawei
300
200
100
0
-100
-200
-300
-400
-500
1500
1700
1900
2100
2300
2500
M+1.96*SD
171.8
M
-115.9
M-1.96SD
-403.5
D-value of predicted and measured values(Unit:Kcal)
Mean value of predicted and measured values(Unit:Kcal)

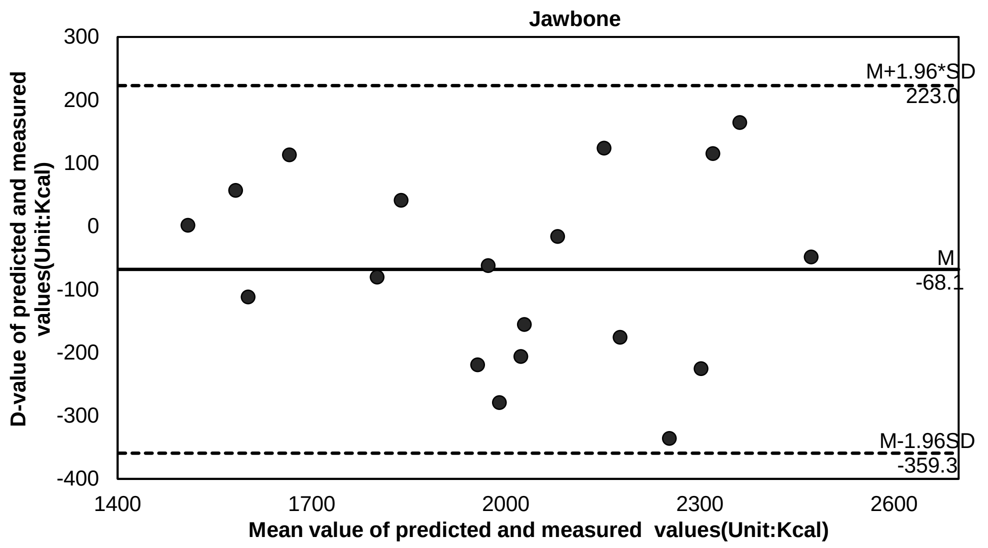
Jawbone
D-value of predicted and measured values(Unit:Kcal)
300
200
100
0
-100
-200
-300
-400
1400
1700
2000
2300
2600
Mean value of predicted and measured values(Unit:Kcal)
M+1.96*SD
223.0
M
-68.1
M-1.96SD
-359.3

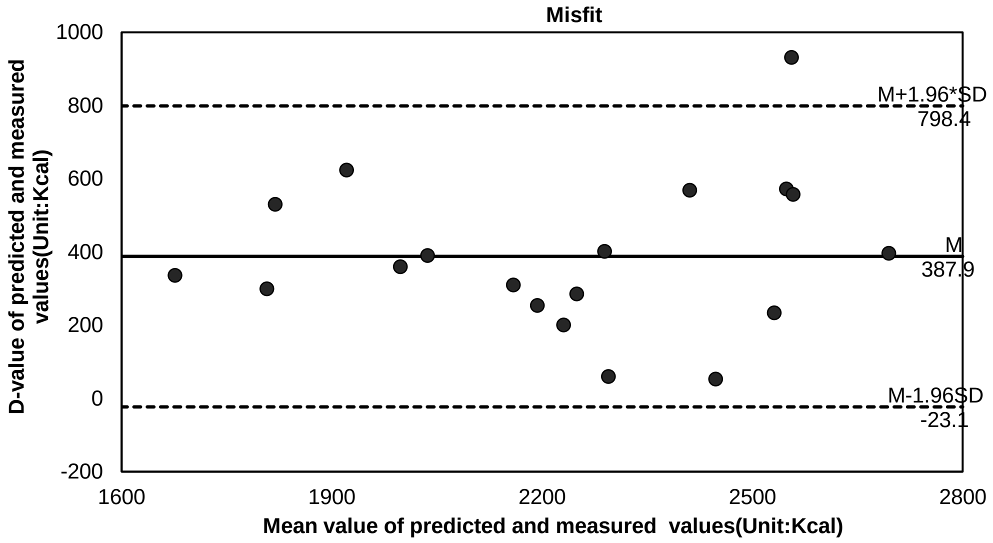
Misfit
D-value of predicted and measured values(Unit:Kcal)
1000
800
600
400
200
0
-200
1600
1900
2200
2500
2800
Mean value of predicted and measured values(Unit:Kcal)
M+1.96*SD
798.4
M
387.9
M-1.96SD
-23.1

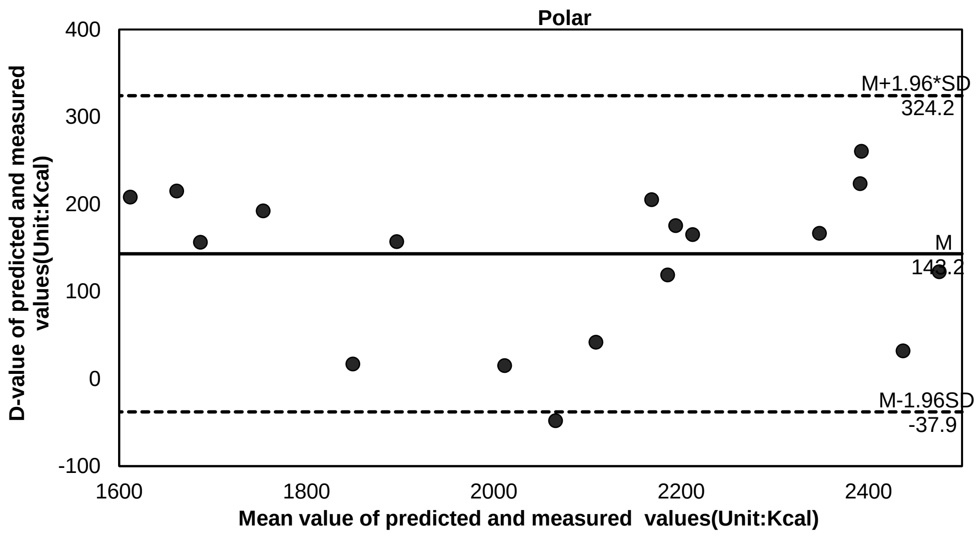
Polar
D-value of predicted and measured values(Unit:Kcal)
400
300
200
100
0
-100
1600
1800
2000
2200
2400
Mean value of predicted and measured values(Unit:Kcal)
M+1.96*SD
324.2
M
143.2
M-1.96SD
-37.9

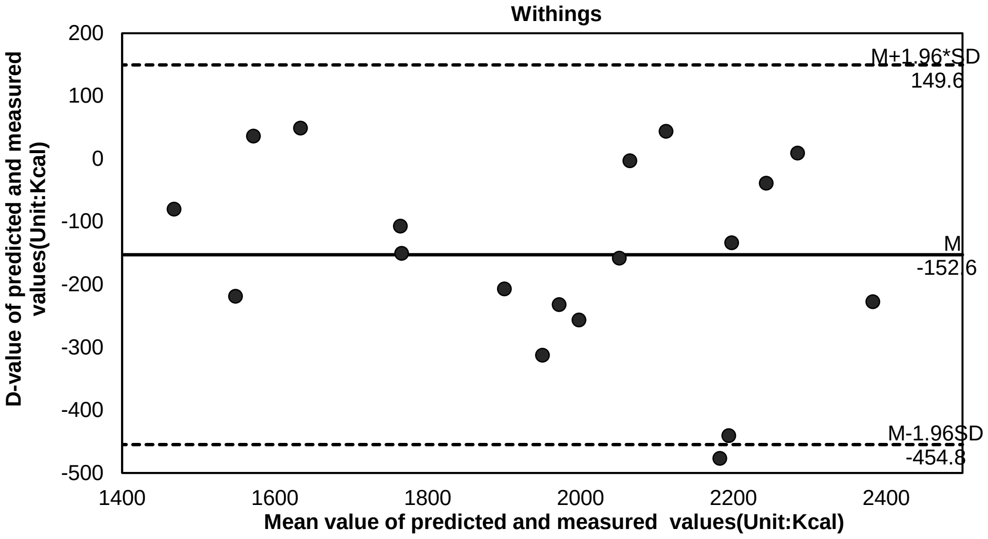


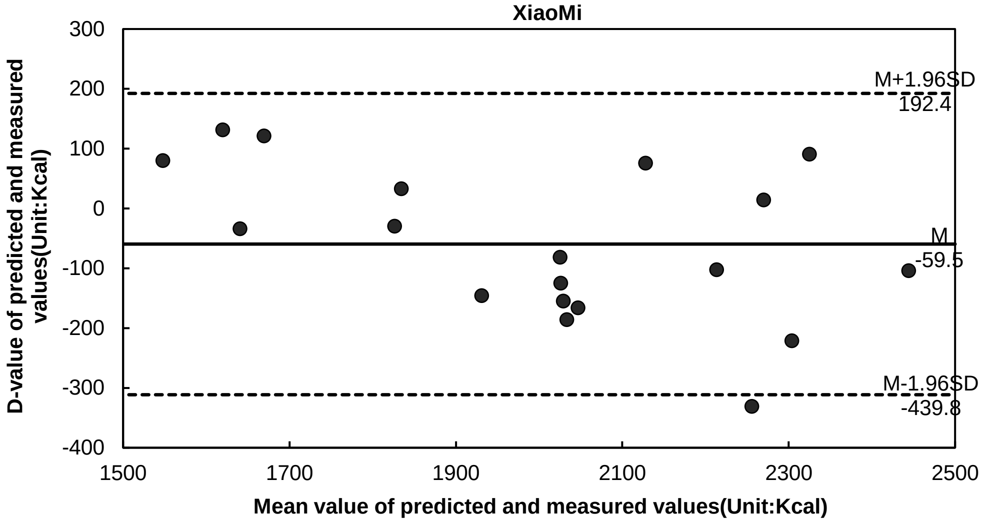


**b**

Figure S1 The Bland-Altman scatterplot of each wrist-wearable activity devices for steps and total energy expenditure.
